# Knowledge, attitudes, and practices of gynecologic oncology nurses toward patient decision aids for fertility preservation: a cross-sectional study in Zhengzhou, China

**DOI:** 10.3389/fpubh.2026.1898056

**Published:** 2026-09-03

**Authors:** Xiaoyu Han, Xiaoxue Li, Manman Li, Tian Tian, Kaige Shi, Yun Shi, Fengzhi Zhang

**Affiliations:** 1 Department of Gynecology, The Third Affiliated Hospital of Zhengzhou University, Zhengzhou, China; 2 Department of Gynecology, Henan Cancer Hospital, Zhengzhou, China; 3 Department of Nursing, The Third Affiliated Hospital of Zhengzhou University, Zhengzhou, China

**Keywords:** knowledge, attitudes, and practices (KAP) study, clinical nurses, fertility preservation, gynecologic cancer, patient decision aids

## Abstract

**Introduction:**

To investigate nurses’ knowledge, attitudes, and practices regarding patient decision aids for fertility preservation in gynecologic oncology departments and to evaluate the associated factors.

**Methods:**

This cross-sectional study used a questionnaire survey and convenience sampling. A total of 201 nurses working in gynecologic oncology-related departments of tertiary first-class hospitals in Zhengzhou, China, were recruited. The questionnaire covered demographic information, knowledge of patient decision aids for fertility preservation, attitudes toward these aids, self-reported practice, and perceived barriers to their use. Data were analyzed using SPSS version 27.0.

**Results:**

Nurses held positive attitudes toward fertility preservation decision aids, but their overall practice level was low. Only 52.8% of nurses frequently or always introduced the existence of these tools to patients; 56.2% never or rarely actively learned about them, and nearly half (49.8%) were unclear about the process for using them. Multiple linear regression analysis showed that reproductive specialist nurse status and years of work experience were independent positive predictors of knowledge scores, whereas an associate degree or below and nurse title were negative predictors. The title of associate chief nurse was positively associated with attitude scores; age and other professiona l titles had no significant predictive effects. Individual characteristics were not associated with practice scores. The main barriers were lack of operational knowledge and lack of training.

**Conclusion:**

Nurses in gynecologic oncology-related departments need to improve their practice regarding fertility preservation decision aids practice, as positive attitudes were accompanied by relatively low reported practice levels. Bridging this gap requires standardized training, improved tool accessibility, and multifaceted efforts.

## 1 Introduction

The incidence of gynecologic cancers is high and has shown a trend toward younger age at onset (1). Cancer treatments often cause irreversible damage to the fertility of patients of childbearing age. Studies have shown that most young patients with gynecologic cancer still have a strong desire for fertility after diagnosis (2). However, decisions regarding fertility preservation involve multiple factors, including medical, ethical, psychological, and economic considerations (3). Patients face pressures such as information asymmetry, time constraints, and anxiety, which can lead to decisional conflict and regret and ultimately affect long-term quality of life.

Fertility preservation decision aids (PtDAs) can systematically present the benefits and risks of each fertility preservation option, helping patients clarify their personal values and make decisions aligned with their preferences. Their effectiveness has been demonstrated in studies in China and internationally (4–6). However, the effectiveness of implementing PtDAs in clinical practice depends heavily on healthcare providers’ level of knowledge and willingness to recommend them (7, 8). As healthcare professionals who have the most frequent contact with patients, nurses play a key role in providing information during the decision-making process (9). Their knowledge, attitudes, and practices regarding PtDAs directly influence the clinical application of these tools.

Whether nurses use PtDAs is essentially a matter of clinical behavior change. In the field of behavior change research, multiple theoretical models have been developed to explain this process. Among them, the Knowledge-Attitude-Practice (KAP) model is one of the most fundamental and widely used theoretical frameworks, positing that knowledge acquisition-attitude formation-behavior change constitutes a progressive pathway, with knowledge and attitude serving as prerequisites for behavior (10).

The KAP model was selected for this study for the following reasons. First, this is an exploratory cross-sectional survey with the primary objective of describing the current status rather than explaining the complete mechanisms of behavior formation. Second, in the Chinese context, systematic data on nurses’ awareness of PtDAs are largely absent, making the KAP model the most parsimonious and direct survey framework to fill this baseline data gap. Previous studies have indicated that the effective implementation of PtDAs in clinical practice is limited by various barriers, such as nurses’ insufficient relevant knowledge, inadequate operational training, lack of institutional support, poor accessibility of tools, and time constraints (11–14). However, existing evidence is derived primarily from Western countries and South Korea, and no systematic data are available regarding Chinese nurses’ knowledge, attitudes, and practices toward PtDAs. Therefore, based on the KAP framework, this study aims to examine the current status of nurses’ knowledge, attitudes, and practices concerning fertility preservation PtDAs in relevant departments, so as to inform the development of training programs and implementation strategies.

## 2 Materials and methods

The study participants were nurses working in the departments of gynecology, gynecologic oncology, and reproductive medicine at tertiary first-class hospitals in Zhengzhou, Henan Province, between November and December 2025. Data was collected online through Questionnaire Star. The inclusion criteria were as follows: (1) possession of a valid nurse practice certificate, (2) employment in a department of gynecology, gynecologic oncology, or reproductive medicine, and (3) more than 1 year of experience in oncology nursing. Nurses who were not on duty during the study period (e.g., those on training, leave, or secondment) and student nurses were excluded.

This study is a cross-sectional survey. According to the requirements for sample size based on factor analysis, the sample size should be 5 to 10 times the number of scale items. The KAP scale used in this study contains a total of 17 items (7 knowledge items, 6 attitude items, and 4 practice items). Using the strictest standard (10 times the number of items), the required sample size is 17 × 10 = 170. Considering the possibility of invalid questionnaires, the sample size was increased by a 20% non-response rate, planning to recruit at least 213 participants. Ultimately, 201 valid questionnaires were collected in this study. Although this is slightly below the expanded target of 213, it exceeds the minimum requirement of 170 (corresponding to 11.8 times the number of items), meeting the sample size requirement for factor analysis. This study was approved by the Ethics Committee of the Third Affiliated Hospital of Zhengzhou University.

The self-administered questionnaire was designed to assess gynecologic oncology nurses’ KAP regarding PtDAs for fertility preservation. It was developed based on the KAP theoretical framework and a literature review. We conducted a pilot test of the questionnaire items among five nurses from a gynecologic oncology department (who did not participate in the main survey). The purpose was to assess the clarity, comprehensibility, and acceptability of the items. All five nurses confirmed that the items were clear and easy to understand, and no major revisions were required. A pretest was conducted with five experts in gynecologic oncology (mean working experience in the specialty, 17.8 ± 8 years; 80% held a master’s degree or higher) through two rounds of expert consultation to test content validity and improve the clarity and interpretability of the items. The questionnaire consisted of three sections. The first section focused on nurses’ sociodemographic characteristics (sex, age, education level, and professional title), certification as oncology or reproductive nurses, years of work experience, department, hospital level, and the monthly number of gynecologic cancer patients admitted to their department. The second section consisted of 17 questions on participants’ knowledge (7 questions), attitudes (6 questions), and practices (4 questions) regarding PtDAs for fertility preservation in patients with gynecologic cancer. Knowledge and attitude questions were evaluated using a five-point Likert scale (1 = completely unaware/strongly disagree, 5 = very aware/strongly agree). Practice questions used a five-point Likert scale (1 = never, 5 = always). The total score ranged from 17 to 85, with higher scores indicating better KAP levels. The scale-level content validity index (S-CVI) was 0.976, and item-level content validity indexes (1-CVI) ranged from 0.8 to 1.0. Cronbach’s *α* coefficients for the knowledge, attitude, and practice dimensions were 0.974, 0.967, and 0.961, respectively. And the split-half reliability of the total questionnaire was 0.83. The third section was a barriers questionnaire consisting of 9 items identified through a literature review and group discussion. Items were rated on a five-point Likert scale (1 = strongly disagree, 5 = strongly agree), with higher total scores indicating greater barriers. Additionally, an open-ended question asked nurses to list other potential barriers encountered in daily clinical practice. The full questionnaire (Chinese original with English translation) is provided as Supplementary Appendix S1.

### 2.1 Statistical analysis

Data were extracted from the questionnaires and analyzed using SPSS version 27.0. Categorical variables were described using frequencies, percentages, or rates. Normally distributed continuous variables were presented as mean ± standard deviation. Between-group comparisons were performed using independent samples t-test or analysis of variance (ANOVA). Multiple linear regression analysis was used to identify factors influencing nurses’ KAP. Statistical significance was set at *p* < 0.05.

## 3 Results

### 3.1 Sociodemographic and other characteristics of the participants

This study recruited nurses from gynecologic oncology-related departments of five tertiary grade A hospitals in Zhengzhou, Henan Province. The characteristics of the participants are summarized in Table 1. The mean age of the participants was 35.26 ± 6.4 years. Among the participants, 99% were female, most held a bachelor’s degree (89.05%), the predominant professional title was charge nurse (68.0%), and 129 nurses had more than 10 years of work experience. Most nurses (68.1%) worked in gynecology departments.

**Table 1:** Characteristics of the study participants (*n* = 201).

Variable	*n*	Percentage (%)
Gender		
Male	2	1.0
Female	199	99.0
Age category		
<30	49	24.4
30~	120	59.7
40~	27	13.4
≥50	5	2.5
Education		
Associate degree or below	4	1.9
Bachelor ‘s degree	179	89.1
Master’s degree or above	18	8.9
Professional title		
Nurse	15	7.5
Primary Nurse	38	19.0
Charge Nurse	137	68.0
Associate Chief Nurse	9	4.5
Chief Nurse	2	1.0
Reproductive specialist nurse		
Yes	15	7.5
No	186	92.5
Oncology specialist nurse		
Yes	31	15.4
No	170	84.5
Years of experience		
1 ~ 5	28	13.9
6 ~ 10	44	21.8
11 ~ 15	73	36.3
16 ~ 20	31	15.4
>20	25	12.4
Department category		
Gynecology	137	68.1
Gynecologic Oncology	25	12.4
Reproductive Department	39	19.4
Number of gynecological cancer patients admitted each month		
≤10	62	32.8
11–20	45	22.3
21–30	20	9.9
>30	74	36.8

### 3.2 Nurses’ knowledge of fertility preservation decision aids

Overall, most nurses had a moderate to high level of understanding of the basic concepts, functions, and effects of PtDAs. For the first six items, the proportion of nurses who responded “somewhat aware” or “very aware” ranged from 72.1 to 77.3%. However, the seventh item showed a relatively lower level of awareness regarding the process for using PtDAs: 10.4% were basically unaware, 5.0% were completely unaware, and another 26.4% were uncertain (Table 2).

**Table 2:** Knowledge distribution of participants regarding fertility preservation decision aids.

Item	Completely unaware	Basically unaware	Uncertain	Somewhat aware	Very aware
Fertility preservation decision aids is an evidence-based standardized tool.	0 (%)	16 (8.0%)	4 (19.9%)	81 (40.3%)	64 (31.8%)
Fertility preservation decision aids are written materials or videos that help patients make better health and medical decisions.	0 (%)	11 (5.5%)	43 (21.4%)	92 (45.8%)	55 (27.4%)
Fertility preservation decision aids are designed to present options based on research evidence and to help patients consider which options best suit their personal circumstances and preferences.	1 (0.5%)	14 (7.0%)	41 (20.4%)	87 (43.3%)	58 (28.9%)
The core functions of fertility preservation decision aids include providing comprehensive medical information (such as success rates, risks, and procedures), clarifying personal values, and generating personalized treatment plans.	2 (1.0%)	13 (6.5%)	38 (18.9%)	90 (44.8%)	58 (28.9%)
Fertility preservation decision aids are effective in improving decision quality and reducing decision regret and Decisional conflict.	0 (%)	10 (5.0%)	46 (22.9%)	81 (40.3%)	64 (31.8%)
Patient decision aids can be used by patients alone, or together with the healthcare team.	1 (0.5%)	15 (7.5%)	49 (24.4%)	79 (39.3%)	57 (28.4%)
I understand the usage process of decision aids.	5 (2.5%)	21 (10.4%)	53 (26.4%)	62 (30.8%)	60 (29.9%)

### 3.3 Nurses’ attitudes toward fertility preservation decision aids

Overall, nurses held positive attitudes toward fertility preservation PtDAs. Specifically, 81.6% were interested in these tools, and 84.6% expressed willingness to discuss them with patients. Moreover, more than 80% believed that the information provided by the tools was scientific and authoritative and that their use in clinical practice helped provide information to patients systematically. Meanwhile, 86.6% were willing to use the tools to communicate more effectively with patients and engage in shared decision-making, and 86.1% were willing to recommend and use PtDAs for patients with fertility intentions (Table 3). Overall, the proportion of nurses who responded “agree” or “strongly agree” to each item exceeded 80%, indicating a high level of acceptance and willingness to use these tools.

**Table 3:** Attitude distribution of participants regarding fertility preservation decision aids.

Item	Strongly disagree	Disagree	Neutral	Agree	Strongly agree
I am interested in fertility preservation decision aids.	0 (0%)	1 (0.5%)	36 (17.9%)	90 (44.8%)	74 (36.8%)
I am willing to discuss fertility preservation decision aids with patients.	0 (0%)	1 (0.5%)	30 (14.9%)	94 (46.8%)	76 (37.8%)
Using fertility preservation decision aids in clinical practice helps to provide information to patients in a systematic manner.	0 (0%)	2 (1.0%)	24 (11.9%)	93 (46.3%)	82 (40.8%)
I believe that the information provided by fertility preservation decision aids is scientific and authoritative.	0 (0%)	0 (0%)	30 (14.9%)	91 (45.3%)	80 (39.8%)
I am willing to use this tool to communicate more effectively with patients and to engage in shared decision-making.	0 (0%)	0 (0%)	27 (13.4%)	87 (43.3%)	87 (43.3%)
I am willing to recommend and use fertility preservation decision aids in my work for patients with fertility intentions.	0 (0%)	0 (0%)	28 (13.9%)	91 (45.3%)	82 (40.8%)

### 3.4 Nurses’ practice behaviors regarding fertility preservation decision aids

Nurses’ overall practice level was low. Introducing the existence of PtDAs to patients was the most common practice behavior (52.8%). However, more than half of the nurses did not use the tools to assist patients in understanding and selecting appropriate fertility preservation measures. Notably, 56.2% of nurses never or rarely actively learned about the tools, and 41.7% never or rarely received relevant training (Table 4).

**Table 4:** Practice behaviors of participants regarding fertility preservation decision aids.

Item	Never	Rarely	Sometimes	Often	Always
I actively learn the relevant knowledge and operational procedures of fertility preservation decision aids.	32 (15.9%)	81 (40.3%)	61 (30.3%)	24 (11.9%)	3 (1.5%)
I introduce the importance and operational procedures of fertility preservation decision aids to patients with medical indications for fertility preservation and their families.	10 (5.0%)	27 (13.4%)	58 (28.9%)	53 (26.4%)	53 (26.4%)
I will use this tool to assist patients in understanding and selecting appropriate fertility preservation measures.	11 (5.5%)	29 (14.4%)	68 (33.8%)	82 (40.8%)	11 (5.5%)
I receive relevant training on how to use fertility preservation decision aids.	21 (10.4%)	63 (31.3%)	76 (37.8%)	39 (19.4%)	2 (1.0%)

### 3.5 Factors associated with the overall KAP scores

The associations between KAP scores and provider characteristics are presented in Table 5. Male nurses had higher knowledge, attitude, and practice scores than female nurses, but these differences were not statistically significant. However, because the sample included only 2 male nurses, these results should be interpreted with caution. Age category was significantly associated with both knowledge and attitude scores. Knowledge scores showed an increasing trend with age, and attitude scores were highest in the 40–50-year age group. Although practice scores also increased with age, the difference did not reach statistical significance. Education level had a significant effect on knowledge scores, with nurses holding a master’s degree or above having the highest knowledge scores. There were no significant differences in attitude or practice scores across education levels. Professional title was also significantly associated with knowledge and attitude scores; associate chief nurses had the highest scores in both domains, whereas practice scores did not differ significantly across titles. Being a reproductive specialist nurse had a significant positive effect on knowledge scores, and although attitude and practice scores were slightly higher, the differences were not significant. Oncology specialist nurses showed no significant differences in any scores. Years of experience were significantly and positively correlated with knowledge scores. Department category had significant effects on both knowledge and practice scores. Nurses in reproductive departments had the highest knowledge and practice scores.

**Table 5:** Overall knowledge, attitude, and practice mean scores and provider characteristics.

Provider characteristics	*n*	Knowledge score		Attitude score		Practice score	
		Mean ± SD	*p*	Mean ± SD	*p*	Mean ± SD	*p*
Sex							
Male	2	28.50 ± 7.77	0.677	29.50 ± 0.7	0.054	14.00	0.105
Female	199	27.38 ± 5.6		25.44 ± 3.86		11.91 ± 2.65	
Age category							
<30	49	25.42 ± 6.25	0.002	24.51 ± 3.28	0.011	11.69 ± 2.82	0.514
30~	120	27.41 ± 5.47		25.40 ± 4.11		11.87 ± 2.66	
40~	27	30.48 ± 4.26		27.55 ± 3.16		12.51 ± 2.21	
≥50	5	29.80 ± 3.03		25.60 ± 3.28		12.80 ± 2.77	
Education							
Associate degree or below	4	17.0 ± 10.41	<0.001	22.75 ± 1.71	0.292	13.50 ± 0.57	0.432
Bachelor ‘s degree	179	27.45 ± 5.64		25.48 ± 3.97		11.87 ± 2.65	
Master’s degree or above	18	29.16 ± 3.91		26.11 ± 2.82		12.22 ± 2.81	
Professional title							
Nurse	15	21.93 ± 4.89	<0.001	25.06 ± 2.93	0.024	12.66 ± 1.79	0.533
Primary Nurse	38	27.11 ± 6.53		25.07 ± 3.91		12.31 ± 2.90	
Charge Nurse	137	27.83 ± 5.24		25.43 ± 3.94		11.78 ± 2.66	
Associate-Chief Nurse.	9	31.55 ± 4.06		29.33 ± 1.41		11.33 ± 2.69	
Chief Nurse	2	25.50 ± 3.53		22.00 ± 2.82		13.00	
Reproductive specialist nurse							
Yes	31	30.93 ± 3.23	0.008	26.45 ± 3.33	0.403	12.58 ± 2.39	0.546
No	170	2675 ± 5.78		25.31 ± 3.94		11.82 ± 2.68	
Oncology specialist nurse							
Yes	15	29.60 ± 5.02	0.513	25.60 ± 4.08	0.890	12.13 ± 2.58	0.524
No	186	27.22 ± 5.69		25.55 ± 3.85		11.92 ± 2.66	
Years of experience							
1 ~ 5	28	27.5 ± 5.49	0.006	25.53 ± 2.79	0.054	11.85 ± 2.73	0.085
6 ~ 1	44	26.18 ± 6.67		24.52 ± 3.84		11.86 ± 2.89	
11 ~ 15	73	27.78 ± 5.09		25.09 ± 4.24		11.43 ± 2.64	
16 ~ 20	31	29.12 ± 5.55		26.87 ± 3.84		13.00 ± 2.41	
>20	25	29.24 ± 4.38		26.52 ± 3.34		12.32 ± 2.65	
Department category							
Gynecology	137	26.61 ± 5.95	0.002	25.63 ± 3.86	0.066	11.92 ± 2.53	0.039
Gynecologic Oncology	25	27.32 ± 4.87		23.84 ± 3.83		10.92 ± 3.36	
Reproductive-Department	39	30.21 ± 4.11		26.00 ± 3.73		12.64 ± 2.35	
Number of gynecological cancer patients admitted each month							
≤10.	62	29.37 ± 5.01	0.001	25.90 ± 3.61	0.482	12.53 ± 2.07	0.150
11–20	45	26.42 ± 5.72		25.37 ± 4.22		11.84 ± 2.80	
21–30	20	24.25 ± 6.20		24.35 ± 3.60		11.20 ± 2.96	
>30	74	27.18 ± 5.54		25.50 ± 3.93		11.70 ± 2.84	

### 3.6 Association between participant characteristics on KAP scores

To identify independent predictors of knowledge scores, stepwise multiple linear regression (entry criterion: *F* ≤ 0.05; removal criterion: *F* > 0.10) was performed with variables that were statistically significant in the univariate analysis as candidate predictors. The final model identified four independent predictors, explaining 17.6% of the variance in knowledge scores (Adjusted *R*^2^ = 0.176, *F* = 11.658, *p* < 0.001). As shown in Table 6, nurses with the title of nurse scored significantly lower than those with the title of charge nurse (*B* = −3.214, 95% CI: −6.175 to −0.253, *β* = −0.149, *p* = 0.034). Reproductive specialist nurses scored significantly higher than non-reproductive specialist nurses (*B* = 3.345, 95% CI: 1.334–5.357, *β* = 0.214, *p* = 0.001). Nurses with an associate degree or below scored significantly lower than those with a bachelor’s degree (*B* = −8.229, 95% CI: −13.504 to −2.955, *β* = −0.203, *p* = 0.002). Years of work experience was positively associated with knowledge scores (*B* = 0.125, 95% CI: 0.021–0.230, *β* = 0.161, *p* = 0.019). Collinearity diagnostics indicated no severe multicollinearity (VIF range: 1.029–1.180).

**Table 6:** Stepwise multiple linear regression analysis of factors associated with knowledge scores.

Variable	*B*	SE	*β*	*t*	*p*	95%CI	VIF
(Constant)	25.676	0.812		31.626	<0.001	24.075–27.277	
Nurse	−3.214	1.501	−0.149	−2.141	0.034	−6.175 to −0.253	1.180
Reproductive specialist nurse	3.345	1.020	0.214	3.280	0.001	1.334–5.357	1.029
Associate degree or below	−8.229	2.675	−0.203	−3.077	0.002	−13.504 to −2.955	1.057
Years of work experience	0.125	0.053	0.161	2.363	0.019	0.021–0.230	1.131

*R*^2^ = 0.192, Adjusted *R*^2^ = 0.176, *F* = 11.658, *p* < 0.001. VIF, variance inflation factor. Reference categories were as follows: Charge Nurse (professional title), No (reproductive specialist nurse status), Bachelor’s degree (education). Only variables that entered the final stepwise model are shown.

For attitude scores, only two variables (age and professional title) showed statistical significance in the univariate analysis; therefore, regression analysis was not performed due to insufficient candidate predictors. For practice scores, no variables reached statistical significance in the univariate analysis, and none of the factors examined in this study predicted participants’ practice scores.

### 3.7 Barriers to nurses’ use of PtDAs

The specific items of the four highest-scoring barriers are presented in Table 7. An optional open-ended question was also included in this study to capture additional barriers not covered by the structured items. However, no participants provided written responses to this item.

**Table 7:** Specific items of barriers to tool application.

Barrier items	Score
Lack of knowledge on how to use fertility preservation decision aids	3.54 ± 1.11
Not knowing where to obtain fertility preservation decision aids	3.51 ± 1.11
Lack of training related to fertility preservation decision aids	3.38 ± 1.12
High costs of fertility preservation limit patients’ use of fertility preservation decision aids	3.34 ± 1.10

## 4 Discussion

Nurses held very positive attitudes toward PtDAs (agreement rate >80% for all items), but their overall practice level was low, and nearly half were unclear about the process for using these tools. The KAP theory suggests that translating attitudes into behavior requires external support. Our study further identified barriers to the application of these tools, including lack of operational knowledge, lack of training, and not knowing where to obtain the tools (Table 7). This indicates that even if nurses have positive attitudes, their practice level is unlikely to improve unless institutions provide training and convenient access to the tools. Our findings are similar to those of studies conducted in other countries. A UK study (11) reported that 87.2% of surveyed participants had never received relevant training, and a Swedish study (12) showed that 40% of nurses identified lack of training as a major barrier. Notably, 85% of nurses believed that short-term structured educational programs would improve practice. A systematic review from South Korea pointed out that knowledge deficits, role ambiguity, and lack of confidence are common barriers faced by nurses (13). These data support the notion that lack of training and lack of operational knowledge may be common issues globally. However, unlike a qualitative study conducted in South Korea that identified role ambiguity as a core barrier (14), nurses in our study more commonly reported problems with tool accessibility. This difference may be attributable to differences in the healthcare systems of the two countries. In South Korea, some PtDAs have been integrated into the electronic medical record system, whereas the hospitals surveyed in our study had not yet established such a support system, making tool acquisition itself a barrier. However, the methodological differences between the two studies may partly explain the divergent findings regarding barrier identification. Based on these findings, tiered interventions are recommended: to address the barrier of how to use the tools, develop standardized instructional videos and quick-reference guides; to address the barrier of not knowing where to obtain them, develop culturally adapted decision-support tools and embed them electronically into the nursing information system to enable one-click access; and incorporate the use of these tools into the responsibilities of oncology nurses along with specialized training. These measures may help resolve the gap between willingness and ability to act.

Multiple linear regression revealed that knowledge scores were influenced by several personal factors. Reproductive specialist nurses had higher scores, which may be related to their greater daily exposure to fertility issues and more specialized training. Nurses with ≥15 years of work experience and those with a master’s degree or above had significantly higher scores than those in other groups, whereas nurses with junior titles had the lowest scores. These findings suggest that accumulated clinical experience and higher education are key facilitators of improved nursing knowledge. Through extensive clinical experience and case exposure, senior nurses may accumulate substantial practical knowledge. Their roles as educators or managers may also motivate them to update their knowledge continuously, and they may have more opportunities for continuing education and professional training, allowing them to access current knowledge of fertility preservation systematically. Lower scores among junior nurses may mainly reflect limited experience and an insufficient knowledge base rather than deficiencies in professional attitude or competence (15). Nurses with a master’s degree or above had the best understanding of PtDAs, which may be attributable to the emphasis of graduate education on evidence-based nursing, research methods, and critical thinking. These nurses may therefore be better able to evaluate and apply such tools. Additionally, graduate students may have stronger self-directed learning and technology adaptation abilities, making it easier for them to recognize the usefulness and ease of use of PtDAs (16).

This study found that only the title of associate chief nurse was positively correlated with the attitude score. Attitudes may be influenced more by subjective factors such as personal beliefs, department culture, and prior training experiences than by demographic or professional characteristics (17, 18). However, given the cross-sectional design, these associations should be interpreted as exploratory rather than causal.

One of the most notable findings of this study is that none of the individual characteristics examined entered the regression model for practice scores. Knowledge scores were influenced by individual factors such as education level and professional title, whereas practice scores were not associated with these factors—this contrast suggests that what determines whether nurses use PtDAs may not be “who they are,” but rather “the environment in which they work (19).”This phenomenon is referred to in implementation science as the “KAP gap” or “knowledge–action gap,” which indicates that knowledge and attitudes do not automatically translate into behavior, but are constrained by external environmental conditions (20). In the absence of systematic training, clear institutional support, and poor accessibility of tools, even nurses with good knowledge and positive attitudes may find it difficult to translate their intentions into actual practice. Individual-level interventions must be combined with organizational-level interventions to effectively promote behavior change. Similar phenomena have been widely reported in the nursing field. In studies on pain management (21), pressure ulcer prevention (22), and evidence-based practice implementation (19), a significant disconnect has been observed between nurses’ knowledge and attitudes and their clinical practice. The core barriers are often not nurses’ own capability or willingness, but systemic constraints such as organizational structure, resource allocation, and management support. The findings of this study further support this view, suggesting that when developing strategies for PtDAs promotion, efforts should shift from simply “training nurses” to “optimizing the system,” including embedding PtDAs into nursing information systems, establishing clear clinical application protocols, and providing convenient access to the tools.

Based on the observed KAP gaps and the barriers identified in this study, several strategies may be considered to enhance nurses’ practice levels (23). Standardized continuing education programs could be developed to strengthen specialized knowledge, and hospitals might consider purchasing or developing standardized PtDAs to improve accessibility. Additionally, encouraging on-the-job degree advancement may help strengthen evidence-based nursing and critical thinking skills. However, as this was a cross-sectional study, the effectiveness of these strategies should be confirmed through future interventional research.

### 4.1 Limitations

This study has several limitations. First, this exploratory cross-sectional study used convenience sampling without *a priori* power analysis; however, the sample (*n* = 201) exceeded the minimum requirement (*n* = 170) based on the 10:1 item to sample ratio rule for factor analysis. Second, all participants were recruited solely from tertiary hospitals in Zhengzhou, Henan Province, which limits the generalizability of our findings to nurses in other regions or healthcare tiers. Future multicenter studies across diverse settings are needed. Third, all practice data were self-reported and may be subject to social desirability bias and common method bias, as all variables were collected from the same source at a single time point. This may have led to overestimation of practice levels. Future studies should consider incorporating objective measures. Fourth, the cross-sectional design precludes causal inferences; the findings are preliminary evidence of associations rather than proof of causality. Fifth, although content validity (S-CVI = 0.976) and internal consistency (Cronbach‘s *α* = 0.974–0.961) were established, factor analysis was not conducted; therefore, the dimensional structure requires future validation. The high α values may also suggest item redundancy, and future research should consider scale refinement.

## 5 Conclusion

Nurses have positive attitudes toward PtDAs but demonstrate low levels of practice. Knowledge scores are influenced by education level, professional title, specialist status, and years of work experience; attitude scores are associated only with the title of associate chief nurse; and practice behaviors are not predicted by individual characteristics. The main barriers were lack of training, insufficient operational knowledge, and difficulty accessing the tools. Standardized training should be strengthened, tool accessibility should be improved, on-the-job degree advancement should be encouraged, and cross-departmental collaboration should be established to effectively enhance nurses’ practice levels.

## Data Availability

The raw data supporting the conclusions of this article will be made available by the authors, without undue reservation.
