# Supplementary material for: Knowledge, attitudes, and practices of gynecologic oncology nurses toward patient decision aids for fertility preservation: a cross-sectional study in Zhengzhou, China
Source: Front Public Health. 2026 Sep 3;14:1898056. doi: 10.3389/fpubh.2026.1898056 (PMC13581698; doi:10.3389/fpubh.2026.1898056)
Supplement: Supplementary file 1 [file Data_sheet_1.pdf]

妇科肿瘤相关科室护士生育力保存决策辅助工具知信行问卷

问卷指导语：尊敬的专家，您好！非常感谢您参与本次调研！本问卷旨在了解妇科肿瘤诊疗中生育力保存（FP）决策辅助工具的认知现状及障碍因素。**决策辅助工具是在患者面临医疗选择时，帮助其了解不同选择方案的详细信息以实现最佳决策的一种工具。**目前生育力保存决策辅助工具有以下几种：（1）决策辅助手册：是帮助患者进行决策的纸质版材料，其内容包含了与癌症和生育能力相关的信息以及关于不同生育选择的讨论，并列出了每个治疗方案的优缺点，参与者可根据自己的倾向对所有的方案进行评级（2）辅助决策网站：以一种便捷的方式帮助患者进行生育力保护决策，通过图形、视频和动画的形式，让一些不愿阅读大量文字的患者也能够获得相应的信息支持。（3）决策树：通过树状图的方式描述了患者决策的路径、每个路径的后果以及患者到达最终目标的多种方式，从而帮助患者准确地权衡不同的方案和结果。本问卷约占用您 5-8 分钟，您的意见对改善临床实践至关重要。本次问卷收集的数据仅用于科研，严格隐私保护制度，特此声明！

一、基本信息

1.您的性别

A.男

B.女

2.您的年龄？（填空）

3.文化程度

A. 大专及以下

B. 本科

C.硕士及以上

4.您的职称是？

A. 护士

B. 护师

C. 主管护师

D. 副主任护师

E.主任护师

5.是否为肿瘤专科护士

A. 是

B. 否

6. 是否为生殖科护士

A.是

B.否

7.您的工作年限？（填空）（）年

8.您所在的科室是？

A. 妇科

B. 妇科肿瘤

C. 生殖科

D. 放疗科

E.其他（科室名称）

9.您所在医院等级

A. 三级甲等

B. 三级乙等

C. 二级甲等

D. 二级乙等

D.其他

10.您科室每月约收治妇科肿瘤的人数？

A. 每月≤10 例

B.每月 11-20 例

C.每月 21-30 例

D.每月＞30 例

二、生育力保存决策辅助工具知信行问卷

请根据您的实际情况选择，在相应的位置打“√”。

题目（知识）	完全不了解	基本不了解	不确定	比较了解	非常了解
我了解“生育力保存决策辅助工具是一种基于证据的标准化工具”。					
我了解“生育力保存决策辅助工具是帮助患者做出更好的健康和医疗决策的书面材料或视频”。					
我了解“生育力保存决策辅助工具旨在根据研究结果提供选项，并让患者考虑哪些选项最适合他们的个人情况和偏好”。					
我了解“生育力保存决策辅助工具的核心功能包括提供全面的医疗信息（如成功率、风险、流程）、进行价值观澄清，并生成个性化治疗方案”。					
我了解“生育力保存决策辅助工具在提高决策质量和减少决策后悔、决策冲突等方面有效”。					
我了解“患者决策辅助工具由患者单独使用，也可以与医疗团队一起使用”。					
我了解决策辅助工具的使用流程。					
**题目（态度）**	**非常不同意**	**不同意**	**中立**	**比较同意**	**非常赞同**
我对生育力保存决策辅助工具感兴趣。					
我愿意与患者讨论生育力保存决策辅助工具。					
我认为在临床工作中使用生育力保存决策辅助工具有助于系统化地向患者提供信息。					
我认为生育力保存决策辅助工具提供的信息具有科学性与权威性。					

我愿意借助该工具与患者进行更有效的沟通和共同决策					
我愿意在工作中向有生育意愿的患者推荐并使用生育力保存决策辅助工具。					
**题目（行为/实践）**	从不	很少	有时	经常	总是
我主动学习生育保存决策辅助工具的相关知识、操作流程。					
在治疗方案确定前，我向具有生育力保护医学指征的患者及其家属介绍生育力保存决策辅助工具的重要性和操作流程。					
我会通过该类工具协助患者理解并选择适合的生育力保护措施。					
我接受过如何使用生育力保存决策辅助工具的相关培训。					

三、生育力保存决策辅助工具应用的障碍因素

**题目**	非常不同意	不同意	不确定	同意	非常同意
我不知道从哪里可以获取生育力保存决策辅助工具。					
我对如何使用生育力保存决策辅助工具缺乏了解。					
使用决策辅助工具会增加我的工作量。					
我平时的工作过于繁忙，没有额外的时间介绍生育力保存决策辅助工具。					
我所在的医院肿瘤科与生殖科之间缺乏顺畅的转诊协作流程。					
我担心使用该工具可能引发患者更多的焦虑或疑问。					
我所在的医院缺乏推广使用决策辅助工具的明确政策或规定。					
生育力保存决策辅助工具内容对患者来说过于专业，难以理解。					
即使有决策辅助工具，高昂的生育力保存费用仍是患者的主要障碍。					
我认为缺乏与生育保存决策辅助工具相关的培训。					

Knowledge, Attitude, and Practice Questionnaire on Fertility Preservation Decision Support Tools for Nurses in Gynecologic Oncology-related Departments

Dear Expert,

Thank you very much for participating in this survey! This questionnaire aims to understand the current status of knowledge and barriers regarding fertility preservation (FP) decision aids in gynecologic oncology practice. Decision aids are tools that help patients understand detailed information about different options when facing medical decisions, enabling them to make well-informed choices. Currently, fertility preservation decision aids include the following types: (1) Decision aid booklets—paper-based materials that help patients make decisions. They contain information related to cancer and fertility, along with discussions of different fertility options, and list the advantages and disadvantages of each treatment option. Participants can rate all options according to their personal preferences. (2) Decision aid websites—these facilitate fertility preservation decisions in a user-friendly manner, using graphics, videos, and animations to provide informational support to patients who may not wish to read large amounts of text. (3) Decision trees—these describe patients' decision-making pathways, the consequences of each pathway, and the multiple ways patients can reach their final goals through a tree diagram, thereby helping patients accurately weigh different options and outcomes.

This questionnaire will take approximately 5–8 minutes of your time. Your opinions are crucial for improving clinical practice. The data collected in this questionnaire will be used solely for research purposes, and strict privacy protection measures are guaranteed. This statement is hereby declared.

Basic Information

1.Gender

A. Male B .Female

2.Your age (____)

3.Educational level

A. An associate's degree or lower B. Undergraduate C.Master's degree or higher

4. Your Professional Title

A. Nurse

B. Nurse Assistant

C. .Supervisor Nurse

D. Associate Chief Nurse

E. Chief Nurse

5. Are you an oncology nurse specialist?

A. Yes B.No

6.Are you a reproductive health nurse?

A. Yes B.No

7.How many years of work experience do you have? (_____) years

8.Which department are you in?

A.Gynecology B.Gynecologic Oncology Department C.Reproductive Medicine Department

9.The grade of your hospital

A.Tertiary Grade A Hospital

B.Grade III, Class B Hospital

C.Class A, Level 2 Hospital

D.Class II, Grade B Hospital

10.How many gynecologic tumor patients does your department admit each month?

A.$\leqslant$10 cases per month

B.11-20 cases per month

C.21-30 cases per month

D.More than 30 cases per month

Knowledge, Attitude, and Practice Questionnaire on Fertility Preservation Decision Support Tools

Please choose based on your specific circumstances.

**Knowledge Items**	**Completely unaware**	**Basically unaware**	**Uncertain**	**Somewhat aware**	**Very aware**
I understand that fertility preservation decision aids are evidence-based standardized tools.					
I understand that fertility preservation decision aids are written materials or videos that help patients make better health and medical decisions.					
I understand that fertility preservation decision aids are designed to present options based on research evidence and to help patients consider which options best suit their personal circumstances and preferences.					
I understand that the core functions of fertility preservation decision aids include providing comprehensive medical information (such as success rates, risks, and procedures), clarifying personal values, and generating personalized treatment plans.					
I understand that fertility preservation decision aids are effective in improving decision quality and reducing decision regret and decisional conflict.					
I understand that patient decision aids can be used by patients alone, or together with the healthcare team.					
I understand the process of using fertility preservation decision aids.					
**Attitude Items**	**Strongly disagree**	**Disagree**	**Neutral**	**Agree**	**Strongly agree**
I am interested in fertility preservation decision aids.					
I am willing to discuss fertility preservation decision aids with patients.					
I believe that using fertility preservation decision aids in clinical practice helps to provide information to patients in a systematic manner.					
I believe that the information provided by fertility preservation decision aids is scientific and authoritative.					
I am willing to use this tool to communicate more effectively with patients and to engage in shared decision- making.					

I am willing to recommend and use fertility preservation decision aids in my work for patients with fertility intentions.					
**Practice Items**	**Never**	**Rarely**	**Sometimes**	**Often**	**Always**
I actively learn the relevant knowledge and operational procedures of fertility preservation decision aids.					
Before treatment plans are determined, I introduce the importance and operational procedures of fertility preservation decision aids to patients with medical indications for fertility preservation and their families.					
I use this tool to assist patients in understanding and selecting appropriate fertility preservation options.					
I have received relevant training on how to use fertility preservation decision aids.					

Barriers to the application of fertility preservation decision aids

Items	Strongly disagree	Disagree	Neutral	Agree	Strongly agree
I don't know where to obtain fertility preservation decision aids.					
I lack knowledge about how to use fertility preservation decision aids.					
Using decision aids would increase my workload.					
I am too busy with my regular work to have extra time to introduce fertility preservation decision aids.					
There is a lack of smooth referral and collaboration processes between the oncology department and the reproductive medicine department in my hospital.					
I am concerned that using this tool may cause more anxiety or questions in patients.					
My hospital lacks clear policies or regulations to promote the use of decision aids.					
The content of fertility preservation decision aids is too professional for patients to understand easily.					
Even with decision aids, the high cost of fertility preservation remains a major barrier for patients.					
I believe there is a lack of training related to ferti					

lity preservation decision aids.					
